# The History of Medicine from Its Origin until the Ninteeth Century. In Two Volumes

**Published:** 1847-04-01

**Authors:** 


### MEDICO-CHIRURGICAL

### REVIEW.

APRIL, 1847.

I. Histoire de la Medecine depuis son Origine jusqu'au XIX. Siecle. Par le Dr. *P. V. Renouard.* Tom. II. pp. 980. 8vo. Paris, 1846.

The History of Medicine from its Origin until the Ninteeth Century. In two Volumes. By *P. V. Renouard,* M.D.

II. Essai sur l'Histoire et la Philosophie de la Chirurgie. Par M. *Malgaigne.* [Bulletin de l'Academie Royale de Medecine, December, 1846.]

An Essay upon the History and Philosophy of Surgery. By M. *Malgaigne.*

Much as we admire the practical tendencies of the medical mind in this country, we feel by no means disposed to approve of that neglect of, or contempt for, the literature and history of the profession, which we have reason to believe are increasingly prevalent. It is true we cannot expect to derive the same amount of instruction which our forefathers believed themselves to have gained from these studies; but it would be a grievous error to suppose that none of this were obtainable. So omnipotent is genius, that even when struggling with the defective information of an early stage of civilization, and surrounded by every obstacle to ordinary progress, it will frequently realize truths whose stamp is universal, and send forth previsions of future improvements which an astonished posterity, aided by the lights furnished by the advanced state of every branch of the accessory sciences, only confirms and chronicles. A great mind cannot traverse any important branch of human knowledge without leaving impressions, the results of its innermost workings or acute spirit of observation, which our arrogance alone prevents our profiting by.

Again, what employment can more suitably fill the leisure time and period of relaxation of the medical man than an examination into the different opinions and practices which have prevailed respecting the diseases and disorders of our frames? than in tracing out the fate of doctrines, the vagaries of the imagination, and the rude efforts of common sense? No man should consider himself educated unless aware of what has been done in the profession he has adopted for his calling in life; and certain it is that if such information were more generally diffused, we should be spared many reproductions of opinions and practices which history has already condemned as erroneous or mischievous; and we should be better able to properly estimate others which, although novel, have in their analogues and fundamental characters been already judged.

From a study like this we may always rise with gratified feelings. We have seen intellects of a gigantic calibre, elaborate reseach, untiring perseverance, ingenious speculation, earnestly, gloriously, and often successfully engaged in the holiest of wars, that against the evils to which flesh is heir to, succouring poor humanity in its frailest moments. To this legion we may with pride say we too belong, and firmly resolve that never shall act or word of ours derogate from the dignity of so noble an art, and convert that which should nearest approach a divine calling, into a mere instrument for the acquisition of wealth.

At no period could such a study be so appropriate as at the present. An unjustifiable scepticism in the powers of our art has been attempted to be raised by some who had better have acquainted themselves with its achievements: but the reader of its history will soon discover that, in spite of the uncertainties which have obscured it, and the ignorance which has impeded it, its career has been progressive, irregularly so, it is true, but still progressive: and will derive encouragement to assist it farther onward notwithstanding the existence of so much public ignorance, the prevalence of charlatanism, and the timid prognostications of pseudo-professors.

For these reasons we have determined laying a copious analysis of Dr. Renouard's two volumes before our readers. It is true, an excellent article upon the History of Medicine, embracing the same period of time, appeared some years since (October, 1831) in the pages of this Review; yet, as the period elapsed since this is a long one, and M. Renouard views his subject in a somewhat different light, we feel certain that our readers will thank us for furnishing them with an account of his excellent work.

The following is his account of the distribution of the subject:—

"I divide into three books or ages the whole period of time which has passed away. The *first* age commences in the infancy of society, as far back as historical traditions extend, and terminates, towards the end of the second century of the Christian æra, at the death of Galen in the reign of Septimus Severus. This lapse of time constitutes for medicine the *foundation* period. The germs of the art of healing, concealed at first in the instinct of man, are insensibly developed, the bases of the science are laid, and great principles are discussed. The human intellect, always impatient, exceeds in its speculations the limits of the known and the possible. Many branches of the art, such as symptomatology and prognosis, are carried to a remarkable degree of perfection.

"The *second* age, which may be termed that of *transition* (extending from the death of Galen to the revival of letters at the end of the 14th century), offers few materials for the history of Medicine. We no longer observe the struggles or discussions between the partisans of different doctrines. The medical sects are confounded with each other. I cannot better depict this epoch than by comparing it to the chrysalis stage of insect life. While nothing appears changed externally, an admirable metamorphosis is operating within, the progress of which is no-wise revealed. The eye of man only perceives the marvel when it has become accomplished.

"Thus, from the 15th century, when the *third* and last age of medicine, that of *renovation*, commenced, Europe offers us a spectacle of which the most brilliant periods of Rome and Athens can give us no idea. A new life seems to have become infused into the veins of the inhabitants of this part of the world. Science, the fine arts, commerce, manufactures, religion, social institutions, all undergo change, or are about to do so. A crowd of schools are opened for the teaching of medicine; and establishments of which the ancients furnished no models are created, with the object of extending to the poorer classes the benefits derivable from the art of healing. The ingenious activity of the modern Christians undertakes everything, and suffices for everything.

"These three great chronological divisions, however, do not suffice for the classification in the mind of the principal phases of the history of medicine; and I have therefore subdivided each age into a small number of sections, easy of retention, which I have termed periods. The first age contains four of these, the second and third each contain two." P. 21.

The following synoptical Table best exhibits these :—

| | | |
|---|---|---|
| I. The Age of Foundation | 1. Primitive or Instinctive Period | Terminating with the fall of Troy, B.C. 1184. |
| | 2. Sacred or Mystic Period | Terminating with the dispersion of the Pythagorean Society, B.C. 500. |
| | 3. Philosophical Period | Terminating at the Foundation of the Alexandrian Library, B. C. 320. |
| | 4. Anatomical Period | Terminating at the death of Galen, A.D. 200. |
| II. The Age of Transition. | 5. Greek Period | Terminating at the Destruction of the Alexandrian Library, A.D. 640. |
| | 6. Arabic Period | Terminating at the revival of Literature in Europe, A.D. 1400. |
| III. The Age of Renovation | 7. The Erudite Period | Comprehending the 15th and 16th centuries. |
| | 8. TheReformatory Period | Comprehending the 17th and 18th centuries. |

"In this distribution of by-gone time, that portion of the 19th century which we have passed through is wanting. I have expressly excluded it, for the following reasons. Is it possible, I have enquired of myself. to unite contemporary history with the same independence of mind and feeling as that of past times? Secondly, is it not fitting, when speaking of living authors, to omit all biographical details? Thirdly, is it not to be feared I might exaggerate the importance of contemporary opinions and discoveries? And fourthly, can we so well seize the general physiognomy of an epoch in the, midst of which we live? All these considerations have led me to fear not being able to treat the history of our own times upon the same plan as the history of anterior periods, and have induced me to terminate with the end of the 18th century." P. 22.

M. Renouard enters upon his task with the determination that his examination of the various doctrines which have prevailed shall be complete and impartial, and to their due consideration he believes collateral illustrations, derived from the condition of the individual promulgating them, and of the countries in which they flourished, and from prevalent systems of philosophy, he believes essential.

"Celebrated physicians not only exert by their writings an influence upon the progress of the science and the consideration in which the art is held; but they also influence these by their oral instruction, their character, and their conduct. Their lives frequently offer models for imitation, and sometimes faults to be avoided. Frequently, also, the early education of a man, and the circumstances amidst which he is placed, explain to us the direction of his genius, and furnish the key to his successes and reverses. For these reasons I could not entirely neglect the biographical details relative to the most famous physicians, especially when such details had some connection with the general history of the art, or comprised some moral lesson.

"The sciences do not progress separately from each other, but proceed, so to say, hand-in-hand, so that it is rare when their progress is not simultaneous. Nevertheless, an exception to this rule presents itself in the history of the human mind in Europe. During the middle ages, theology and dialectics were cultivated with success, while other branches of human knowledge, and especially medicine, vegetated in the lowest degradation. But after the 14th century, industry, the sciences, and the arts awoke from their prolonged sleep. On the one hand, the civil and political organization of European nations became regulated and their material well-being augmented; and, on the other, the intellectual and moral faculties of the individual members of society became more developed, and thought took a freer, a bolder, and a better direction. It seems to me that the historian would fall short of one of his obligations if he did not, from time to time, cast his eye upon the general condition of society; and therefore, at the commencement of each of my chronological periods, I have given a rapid sketch of the aspect which civilization then put on.

"Another very remarkable fact, and one of capital interest in the history of medical theories, is that they are all derived in a manner more or less immediate from some system of philosophy. Thus, one would have but an incomplete notion of these theories if we were ignorant of the philosophical source whence they have sprung. But we must not attach too much importance to these analogies, nor pretend to judge the value of medical theories by them. It must not be forgotten that a philosophical system may be false in its generality, and yet true in the particular application of it which is made to medicine. In like manner, from an irreproachable philosophical system, we may deduce by false reasoning an erroneous medical theory. Therefore, after having indicated the philosophical ideas with which any medical doctrine seems to be allied, we shall examine this by itself in relation to its practical consequences." P. 11.

M. Malgaigne, in like manner, in his Essay on the Progress of Surgery from the time of Hippocrates, lays great stress upon the extent to which this was favored or impeded by its connection with the various prevalent philosophical systems. He divides the space of time into six periods, and thus designates them:—1st Period, The Socratic Philosophy and Hippocrates. 2nd. The Platonic Philosophy and Galen. 3rd. The prevalence of the dogmas of authority; the surgery of the Lower Empire, and of the Arabs. 4th. The Reform of Luther and Ambrose Paré. 5th. The Philosophy of Descartes and J. L. Petit. 6th. The Philosophy of Bacon and John Hunter.

### I. The Age of Foundation.

[Extending from the Origin of Society to the End of the Second Century after Christ.]

### 1. *The Primitive Period.*

[Of a different duration among various nations.]

M. Renouard judiciously avoids imitating Sprengel and other writers in dwelling at great length upon the earliest and mythological portion of the history; and we need not follow him even in the cursory account he gives of the state of the Art of Healing among the Egyptians, Hebrews, early Greeks, Hindoos, and Chinese. The materials are too scarce to admit of any authentic narrative being deduced, and an excursion into the realms of fable and imagination, however interesting to the poet and metaphysician, would only be to divert the attention of the medical enquirer from more profitable paths of enquiry. For this reason, we need not advert to the short account which M. Renouard gives of Æsculapius and his disciples, merely stating that he considers Machaon and Podalirius to occupy the confines which separate mythology from history—their biographies offering a mixture of fabulous tales and probable relations. Homeric song, by the honorable portion it assigns them at the siege of Troy, places their actual existence beyond a doubt; but their filial relation to Æsculapius, himself in all probability a fabulous character, is indeed problematical.

### 2. *The Mystic or Sacred Period.*

[From the taking of Troy, B. C. 1184, to the Dispersion of the Pythagoreans, B. C. 500.]

Whatever may have been the reality of the existence of Æsculapius, certain it is that *temples* dedicated to his worship were spread over the whole of Greece, and were found in Asia, Africa, and Italy. The priesthood attached to them, under the name of Asclepiades, or descendants of Æsculapius, formed a separate caste, governed by secret statutes. The temples were located on the healthiest sites, surrounded by cheerful vegetation, and resorted to by the sick from all parts. Appropriate regimen, pure air, and a lively faith in the recorded miracles, doubtless frequently achieved a cure; but, while the priesthood surrounded their votaries with every appliance calculated to make a notable impression upon the imagination, they likewise had recourse to special remedies suitable for the various diseases; and prescribed, on occasion, bleeding, purging, vomiting, sea-bathing, and the various therapeutical resources accessible to them. Although bound to secresy by their vows, the priests consented to reveal their practices to those who qualified themselves for the disclosure by initiatory observances; so that at each temple, in all probability, a kind of medical instruction was going on. Of all these temples that at Cos has enjoyed the greatest celebrity from the number of illustrious physicians it formed within its walls. Among the sources of instruction special to this period were the votive tablets, suspended upon the columns and walls of these edifices, and indicating the name of the patient, the disease he was the subject of, and the means employed for its removal. These were probably of little service beyond the re-assurance they imparted to the worshippers: and it is presumable that the Asclepiades prepared in private a more detailed account of the cases which came under their notice; for a long-continued habit of observation and description can alone account for the excellent taste and precision characterizing some of the Hippocratic books.

During the Mystic Period, *Therapeutics* were based on no form of reasoning, Hippocrates, Celsus, and Galen all agreeing that, prior to the introduction of Philosophy into the science of medicine, that is prior to Pythagoras, empiricism exclusively prevailed. "Not a rational empiricism, however, which only took its rise much later in the Alexandrian School, but the natural, instinctive empiricism, such as is followed by persons ignorant of the medical art, when they proffer their experience of the virtues of certain remedies in given diseases. Indeed, gross as the reasoning of such persons may seem, it is yet based upon an incontestible principle which may be thus laid down. Any remedy which has cured a disease ought equally cure diseases resembling the first one." The difficulty here is to establish the positive identity of the diseases in question, however much in some points they may resemble each other; and, just in proportion as our diagnosis becomes more perfect, we shall be in less danger of confounding morbid states essentially different, and better able to separate them from others which they only superficially resemble. The importance of improving our means of diagnosis has attracted the attention of eminent practitioners in all ages; but the mere collections of symptoms, however large, made by the Asclepiades, were manifestly inefficient to this end. The following is M. Renouard's recapitulation of his account of the Mystic Period.

"During the space of about 800 years which this historical period embraces, medicine underwent in Greece its earliest transformation. From being domestic and popular it became sacerdotal, surrounding itself with the apparatus of mystery. Up to that time, princes, captains, and even shepherds, acquired a reputation for skill in this art: but, after the siege of Troy, we only hear of consultations held in the name of the Divinity, in temples or some celebrated caverns. Not that there were not even at this period men besides the priests who intermeddled with the treatment of disease and sold remedies; but scientific medicine, if we may so term the limited knowledge then possessed, was confined to the sacerdotal class, among whom alone it was perpetuated by uninterrupted tradition, and slowly developed amidst darkness and silence.

" 'This practice of medicine in the temples of Æsculapius,' says M. Gauthier, 'may be divided into two epochs. In the first, which extends, down to Hippocrates, the Asclepiades, although for the most part employing but superstitious remedies, rendered good service to science by the taste which some of them manifested for observation. It must be admitted that, in a barbaric period, medicine would make more progress in the hands of a corporation like the Asclepiades, than if it had continued domestic and popular. It was hardly probable that, at so remote a period, when art and science were yet in their infancy, a man of genius would suddenly appear, who would elevate medicine to the rank of a science. In the second epoch, which extends from the time of Hippocrates to the Christian æra, the medicine of the Temples gradually degenerated, and consisted generally in but a gross jugglery. * * * * * * It is difficult at the present time to appreciate the amount of information possessed by the priests of the Temples, and the progress made by medicine in their hands. As there have always been found persons who have shewn a tendency to admire all that is ancient, it is not surprising to find, both among the ancients and moderns, writers who have vaunted beyond all measure the Asclepiadal medicine. On the other hand, some writers deny that they possessed any information whatever. Thus, M. Malgaigne is desirous that the Asclepiades should retire to that obscurity from which they should never have been withdrawn, and proposes to erase them from the history of surgery and medicine. We believe both these extremes are erroneous. It is probable that the perusal of the inscriptions in the temples, and the custom of seeing a great number of sick there, eventually conferred a certain amount of medical knowledge upon the priesthood.'

"This seems to me the most rational conclusion concerning this very obscure portion of the history of medicine. When authentic documents are wanting the imagination allows itself a free course, and upon this occasion, that of the erudite has not been sterile. But in the midst of the contradictory opinions which have been entertained upon the real amount of knowledge of the Asclepiades, that held by M. Gauthier, I repeat, seems to me the most probable and the best founded.

"At last we are now arriving at an epoch in which the art of healing undergoes a new metamorphosis far more interesting for the historian and the philosopher, and far more advantageous for humanity. Until this period, the edifice of medicine had been constructed of materials taken at hazard, and usually collected together without taste or method. No idea of arrangement or premeditated design had directed the researches of the men who made the earliest discoveries. But, in future, reason and genius extend and perfect that which chance and instinct had but sketched out. The scientific monument of this difficult art is about to rise grand and majestic, gradually harmonizing in all its parts. We shall follow its progress through the various periods, no longer by the aid of vague conjecture, but by the succour of authentic documents, and of more or less faithfully preserved remains. We shall no longer be reduced to divine the innermost thoughts of the labourers, at the different phases of its elevation. We shall read this imprinted in intelligible characters upon the fragments of their works which have descended to us." P. 111.

### 3. *The Philosophic Period.*

[From the Dispersion of the Pythagoreans, B. C. 500, to the Foundation of the Alexandrian Library, B.C. 320.]

In this period the priesthood yield the scientific sceptre to the philosophers, thus divesting it of much of the mystery with which it had been surrounded. Hippocrates traces with great sagacity the various circumstances derivable from climate, manners, and political institutions, which serve to explain the progressive character of European as contrasted with Asiatic knowledge; and he deems the Hellenic race at the epoch at which we have arrived, as placed in conditions especially favourable for the development of their moral and physical faculties. The vestiges of the ancient Indio-Egyptian civilization, which formed the earliest model of that of Greece, insensibly disappeared; and the sages of the country, so far from having to wander into distant lands in quest of information, were enabled to render their own country a centre for the enlightenment of the rest of mankind.

M. Renouard furnishes us with brief descriptions of the life and doctrines of Pythagoras, and of the foundation and dispersion of the Italic sect; but, as these have very slight bearing upon the history of medicine, we pass at once to his account of the works and labours of

*Hippocrates.*

Hippocrates was born in the island of Cos, about the year B. C. 460, of a family in whom the practice of medicine had long been hereditary, professing, as it did, to trace its descent direct from Æsculapius. Few particulars of his life are known, and the epoch of his death has been very variously stated. He was, however, a cotemporary, and somewhat younger than Socrates, and flourished in that celebrated Periclean age, when art and science attained their highest splendour in Greece. The temple at Cos being the most celebrated of those dedicated to Æsculapius, he was surrounded with favourable opportunities; but, not content with these, "he traversed also the principal Greek towns of Europe and Asia, discoursing with philosophers, visiting the gymnasia (then become schools of medicine), giving his services to those who sought them, and everywhere accumulating observations, not only upon particular diseases, but upon epidemic constitutions, the influence of manners, climate, regimen, &c." Knowledge so amassed and displayed in his immortal works, procured for him that admiration of his cotemporaries which posterity has so amply confirmed. His children and grandchildren pursued the same profession, and unfortunately affixed his name to the works which they produced, so that, even shortly after his death, it became a matter of difficulty to distinguish what was really his. This difficulty increased with time, both by reason of the ignorance and carelessness of copyists and the trickery of the vendors of books, who, according to Galen, did not scruple to attach the name of Hippocrates to works composed long after his death. The large prices paid for works bearing his name, especially at the period of the formation of the Alexandrian and other libraries, gave encouragement to this fraudulent procedure. An immense number of commentators, from Galen downwards, have occupied themselves in distinguishing the true from the apocryphal writings, and M. Littré, in the introduction to his new translation, enters into the question at great length and with much ability. M. Renouard only accepts as the legitimate productions of the Hippocratic pen, the writings which the principal critics are unanimous in allowing as authentic. "These are the Essay on Prognosis; some of the Aphorisms; the 1st and 3rd books of the Epidemics; On Regimen in Acute Diseases; On Air, Water and Places; On the Joints and Luxations; On Fractures, and the Instruments of Reduction. These do not comprehend a fourth part of the entire collection; but, even so reduced, if judged of by the period in which they were composed, they amply justify the enthusiasm of cotemporaries and the admiration of posterity."

M. Littré has shewn that the "Hippocratic Collection" of works, as known to us, could not have been entirely published prior to the foundation of the great libraries at Alexandria and Pergamus. The major portion of the writings remained in the hands of the successors of Hippocrates, who only communicated them to their disciples. This collection is formed of various fragmentary portions contributed by successive authors, extending over the whole of the period here termed Philosophical—from Pythagoras to Aristotle. As the earliest authentic monument of medical science the collection is worthy of the attention which its intrinsic merits should also command for it, and M. Renouard furnishes a succinct review of the condition of medical science as illustrated by it.

*Anatomy* was necessarily but little known, as neither Hippocrates or his descendants ever dissected the human body, a skeleton, however, being said to have existed at Cos. *Physiology*, in the sense we understand it, as explaining the functions of the various organic apparatus, must necessarily be a dead letter to those ignorant of the structure of these. While ignorant, however, of the uses of the muscles, tendons, nerves, &c. these writers indulged in the most transcendental speculations upon the nature and seat of the vital principle. The subject of *Hygiene* is dwelt upon in the books upon Air, Water and Localities, on Regimen, and on Healthy Diet, and that as completely as the information of the period admitted of.

*Pathology.*—During the period we are engaged in, the animal economy was regarded as an indivisible whole, and the various morbid phenomena as expressions of a general disturbance of the organism rather than of any of its particular portions. Thus, the study of symptoms was pushed to a great extent at Cos, giving rise to the branch of pathology termed *Semeiology*. More than one-eighth part of the entire Hippocratic collection is devoted to this subject. Prognosis embraced what we now divide into diagnosis and prognosis, and being based upon the observation of superficial phenomena not on a knowledge of the condition of internal organs, excited the exertion of much sagacity, and became a frequent cause of error.

"He who is in the habit of seeing patients, who knows from experience the indescribable variety and the inconstancy of morbid symptoms, can alone appreciate the time, labour, and patience requisite for the deduction of certain general propositions from the observation of these phenomena; for the tracing, in a word, these semeiotic rules which the ancient medicine has transmitted to us, and some of which yet retain all their value. If more perfect and more numerous means of investigation now enable us to extend our observation farther, we must not the less admire the perspicacity of the ancients, who, in many cases, were enabled to foresee the future events of diseases with as much certainty as ourselves." P. 149.

*Nosography* was exceedingly defective at this remote epoch, for although the division of diseases into sporadic, epidemic and endemic, met with in several parts of the Hippocratic writings, is well founded and of great utility; yet the distinction between acute and chronic diseases is frequently confounded or not observed. Few of the latter, indeed, are described, and some not even named—regarded, as they were, as inconveniences to be endured rather than as morbid affections susceptible of cure. Even of the acute diseases the descriptions are usually too defective to allow of their specific identification. In *Therapeutics*, the reputed axiom, *contraria contrariis curantur*, which was afterwards so frequently attempted to be elevated into a fundamental principle upon which the medical art was to be based, and has of late been so injudiciously dwelt upon as a rallying cry against homœopathy, obtained a general vogue; not but that some even of the Hippocratic writers already furnished arguments for contesting its soundness, as we shall hereafter see. The works of the Hippocratic collection treating of *Surgery*, which have descended to us, are manifestly imperfect, several subjects being entirely omitted and others only enumerated. Some of these which we possess, as the Treatises upon the Surgeon's Officinum, upon Fractures, and upon the Articulations, are pronounced by M. Malgaigne as unsurpassed by any writings anterior to the 19th century. Several treatises of the Collection relate to *Obstetrics,* and the one on Superfœtation furnishes an excellent account of the art of the accoucheur at this period. Midwives would seem to have been the persons habitually employed, male practitioners only being called in upon emergencies.

As a *clinical instructor,* Hippocrates eminently possessed that moral frame of mind which M. Bouillaud justly insists should be the portion of him who desires to impart the knowledge he has acquired to others. Animated with the sole desire of benefiting his fellow-men, he cheerfully imparts all he knows, and candidly confesses the faults and errors he may have comcommitted. The first and third books of the *Epidemics* present the earliest specimens of clinical collections; and have formed an admirable model for future investigators of medical constitutions. They contain the histories of forty-two cases; but, unfortunately, the regimen and treatment adapted for these are not specified. The other five books of the Epidemics, not attributable to Hippocrates, contain a miscellaneous collection of cases and observations on every variety of disease, in some of which however the treatment employed is detailed.

Upon the celebrated "*Aphorisms,*" M. Renouard offers the following criticism.

"No medical work of antiquity has enjoyed so colossal a reputation as this. Physicians and philosophers have possessed a veneration for it equal to that entertained by the Pythagoreans for the golden verses. Long were the Aphorisms regarded as the capital of the scientifie edifice of medicine, as the sublimest effort of medical genius. Only a few years since, the Faculty of Paris required that candidates should insert a certain number of them in their theses, and probably nothing short of political revolution in France would have been able to discard this old remnant of superannuated worship.

"For some of the propositions which express general truths of recognized utility or delicate and profound appreciations, how many are there which only represent exceptional truths, vulgar reflections, or even errors and contradictions! In a practical point of view, the Aphorisms seem to me almost an absolute nullity, since, maintaining no connexion with each other, they make only a temporary impression upon the mind of the reader, and are quickly effaced from the memory. Besides, admitting that he had them at his finger's ends, the practitioner would be little aided by them in his treatment of disease. This study then can offer no solid advantage to the student, and is fitting only for the practitioner whose experience has ripened his judgment; for he alone can distinguish the true from the false, the good from the bad, in these general maxims. For him alone they form the summaries of a crowd of different and scattered observations." P. 175.

*The Hippocratic Theories and Systems.*—M. Renouard examines these at length, but we have only space for some notice of their leading points. That of *Coction* and *Critical Days* has maintained its ground, with certain modifications, even to our own times. Disease was regarded as a series of phenomena consequent upon the efforts made by the vital principle while operating the coction of the morbigenous matters. These matters could only be expelled the economy after they had attained by such coction a fitting maturity, that is after their elements separated and mixed with the natural humours of thesystem, were re-united into an excrementitious material or humour. The approaching maturity was manifested by a certain aggravation of the symptoms constituting the critical signs which the physician must be able to discern, and respect. The coction operated, the morbid matter has to be evacuated by one of the emunctories, and Nature may herself be equal to such critical discharge, or exhausted by her prior efforts, she may require the intelligent aid of the practitioner, who must direct the matters by appropriate medicaments to such emunctory towards which they naturally tend. If coction be not effected, or be imperfectly accomplished, the vital principle may be overcome by the morbid elements and the patient succumb, or new efforts at coction are set up. The period of days required for the elaboration was termed *critical.* Four days were regarded as the most perfect period, then three, while the seven days attained by adding the ten, has always enjoyed much consideration. A complete scale of such days was indeed formed by adding the numbers three or four to successive periods. To this progression, evidently derived from the Pythagorean doctrine of numbers, Nature, however, frequently refused to subject herself, so that the mode of computation had more than once to be changed, and gave rise to much difference of opinion among the ancients. Notwithstanding these discrepancies, the doctrine of expulsion of morbific matter on critical days has descended to our times; and no one who has attentively studied the histories of the periodical febrile and eruptive diseases will probably be disposed to deny that it possesses some foundation in truth. It is to be remembered that the observations of the Hippocratic writers chiefly related to febrile epidemics; and their error has probably consisted in extending to, and generalizing for all diseases what legitimately can only be applied to a few. Conjoined with this doctrine of coction and crises (and together constituting the original form of *Dogmatism*) was the theory of the *four elements* or elementary qualities, heat, cold, dryness, moisture, and the four *cardinal humours,* the blood, bile, atrabile, and phlegm. When Hippocrates wrote, the natural philosophers had just developed their doctrines of the homogeneity of matter, and the production of its diversity of appearance by the various combinations of its four elementary modes of existence, fire, air, earth, and water; and upon this doctrine he doubtless founded his view of the condition of the animal economy. Speaking of the humours, in his Nature of Man—he says,

"The body of man contains blood, phlegm, and two sorts of bile, yellow and black. Such is its nature, and through these is it well or ill. It is in health when each of these exists in its just proportion of quantity and strength, but especially when they are well commingled. It is ill when any one of them is in excess or deficiency, or is separated in an unmixed state; for when so separated, not only must the part in which it is defective suffer, but likewise that upon which it is cast, becoming overloaded, will suffer pain and irritation." P. 193.

He states that phlegm, being of a cold nature, is most increased in Winter, blood in Spring, bile in Summer and atrabile in Winter.

"Whatever opinion we may form of this system in possession of our actual knowledge, we are compelled to admit that, united to the theory of coction and crises, it offered a plausible interpretation of the physiological phenomena which most attracted the attention of observers at that period. In fact, these phenomena, whatever they may be, can only be produced by an action compounded of vital and of physico-chemical forces, which act simultaneously on the animal economy. Now, the theory of coction expounds the laws according to which the vital principle is supposed to exercise its activity, and that of the elements and humours exhibited, the influence of inorganic forces, and the laws according to which such influence is exerted upon organic bodies. These two theories together constituted the *Ancient Dogmatism;* the original doctrine of the School of Cos, and of which Hippocrates is regarded as the principal author. In this doctrine the humours play the part of physiological or secondary elements—agents endowed with different and even contrary properties, placed at the disposition of the vital principle, which alone impresses upon them a good or bad direction. But these agents may sometimes contract deleterious properties, by reason of the action of external forces not submitted to the vital principle. * * *

"In spite of its errors and imperfections the doctrine of Dogmatism is the most ingenious and complete of all those of antiquity. It better responded than any other to the necessities and tendencies of ancient science, and was therefore received with admiration and adopted, not only by the generality of physicians, but also by the greatest philosophers." P. 197.

M. Renouard protests against two opposite opinions that have been entertained respecting Hippocrates, it being deemed by some that it was his glory to have separated medicine from philosophy, and by others, that this consisted in the intimate union of these, which he effected.

"It is certain that others philosophized before Hippocrates, and equally so that Hippocrates philosophized like others, and that he partook of the prejudices of his age in many respects. But in other respects also his philosophy is distinguishable from that of his cotemporaries by its greater wisdom and depth. He incessantly reminds philosophers and physicians of the maxim, too often neglected by them and sometimes by himself, *that we cannot well inform ourselves of the nature of man without the aid of medical observation, and that we ought to affirm nothing concerning this nature but after having acquired a certainty of it by the evidence of the senses*—a maxim diametrically opposed to the dogmas of Pythagoras, and one that encloses the germ of an entire philosophy, which Plato mistook, and which Aristotle only caught a glimpse of." P. 198.

Upon this subject M. Malgaine observes:—

"When Hippocrates appeared, Socrates had proclaimed a rational method. Philosophy had bestirred itself and found a leader, and Medicine had only to follow the impulse. The characteristic of the Socratic philosophy is the rejection of mere speculation, and the admission only of ascertained facts and positive premises as the basis of all science and reasoning. Therefore he set little store on those researches which tend to no useful and practical end; in fact, above all, Socrates attached himself to three things—*reality, morality,* and *utility.* Xenophon reports to us a fragment of a conversation he had with his master upon medicine; and these few words so distinctly contrast with preceding methods, and so well accord with the Hippocratic manner, that we may here discern the germ of the most brilliant works of the latter. * * * * * You will observe the same method in the masterpieces attributed by general consent to Hippocrates. Everywhere the same love of the real, the same search for the useful, and the deep sentiment of morality which the Hippocratic Oath so admirably expresses. Occasionally, also, we must admit there is some abuse of dialectics, and more than one writer has already signalized, in the discussions into which Hippocrates sometimes enters, the Socratic irony. There is truly the same force in the disciple as in the master, but there is also the same weakness: and indeed, to recapitulate my entire opinion, Hippocrates was the Socrates of medicine. Unfortunately both the one and the other had too far anticipated their age, and their school was not long in becoming corrupted. Socrates, in his old age, naturally was astonished at the language which Plato, the most illustrious of his disciples, attributed to him; and the pale successors of Hippocrates almost immediately quitted the path he had traced out to follow in the train of that philosopher."—*Bulletin*, t. xii., p. 160.

Among the followers of the Hippocratic or ancient (so called probably from its comprising the dogmas most anciently professed in medicine) dogmatism, the celebrated philosophers, Plato and Aristotle, were those who most influenced its progress by the ingenuity or profundity of their speculations. Plato did infinite mischief to the cause of true progress by diverting attention from simple observation, which he despised, to speculations upon the final causes of phenomena. The imagination usurped the province of the senses, and the most visionary speculations were substituted for true scientific procedures.

Aristotle was the most assiduous and constant of the crowds of scholars who resorted to Athens to learn philosophy from the lips of Plato; but, becoming engaged afterwards in researches which have justly stamped him as one of the greatest of naturalists, he soon discovered the fallacy of the *à priori* reasoning then in vogue, and laid down the famous axiom, destined to such ample development, many centuries afterwards, at the hands of our own countrymen Bacon and Locke, that all ideas are derived from the senses, "*nihil est in intellectu quod non priùs, fuerit in sensu.*" M. Renouard exhibits at considerable length the reasons why this great man, who thus conceived the idea of a true philosophy, was prevented deriving the practical consequences which should emanate from the discovery of so valuable a principle. The beneficial tendency of the above-named axiom was counteracted by the operation of another, which declared that "the first ideas we derive from our sensations are *general* ones." After contrasting this with the theory of Locke, M. Renouard observes—

"These two quotations, the one from the writings of Aristotle, the other from those of Locke, offer the curious spectacle of two metaphysicians, setting out from the same principle, that all ideas are derived from the senses, and immediately after separating into widely divergent paths; the one affirming that the first ideas derived by our mind through the interposition of the senses are *general* ones; the other, that they are always *individual* ones. But it is easily seen that, in the examples furnished by Aristotle, he confounds obscure, vague, and indeterminate ideas with general ideas, a grave error scarcely conceivable on the part of such a logician. * * * * * * * From the instant this philosopher believes he has indisputably proved that general ideas are those first formed by the human understanding, he deduces the conclusion that we should commence the study of every science with certain generalities or axioms, which on this account he terms principles, or elements, and thence pass on to the particular or individual ideas or phenomena. So that, after having assigned to our ideas an origin quite opposed to that assigned by Plato, he counsels the very same didactic method or mode of acquisition as that philosopher." P. 249.

M. Renouard pursues at considerable length his consideration of the fanciful ideas on physiology entertained by the strong mind of the Stagyrite, seduced from the true path of observation he had himself indicated and then lost sight of. These are invested with considerable importance in consequence of their partial adoption by Galen, through whose influence they have retained an undue importance until quite modern times. We have, however, not space for the exposition nor for the well-merited eulogia which our author passes upon the vast acquisitions of the great peripatetic philosopher in natural history—acquisitions which were done ample justice to a few years since by Professor Owen in his lectures at the College of Surgeons, with the praiseworthy enthusiasm of a kindred spirit, and a felicity of expression and illustration none who heard can have forgotten.

The following are M. Renouard's concluding observations upon the Historical Period, the consideration of which we have now completed.

"Hippocrates forms the transition between the preceding period and the present, belonging equally to mythology and history; for if some circumstances of his life and some of his works are authentic, most of them are doubtful or controverted. His doctrines have been received by cotemporaries and posterity with a veneration which approaches a worship, less perhaps on account of their real merit than the mystery which surrounded their birth. Since his time no man has obtained, as a physician, so great, so constant, and so universal a homage. Soon indeed anarchy was introduced into the bosom of the school he had rendered so celebrated. Multitudes of methods and theories were there surreptitiously propagated, under cover of his name and authority, so that at last it became impossible to discover, amid so many writings and facts placed to his account, what really belonged to him. After some years of confusion, we shall find physicians divided into three principal sects, which, struggling against each other with varying success, eventually become absorbed by the most powerful." P. 260.

### 4. *The Anatomical Period.*

[From the Foundation of the Alexandrian Library, B. C. 320, to the Death of Galen, A. D. 200.]

*The School of Alexandria.*—Among the lieutenants who dismembered the vast empire left at their mercy by the death of Alexander, two only, Eumenes, governor of Pergamus, and Ptolemy-Lagus, viceroy of Egypt, turned their attention to the civilizing effects of literature. The foundation of the libraries at Pergamus and Alexandria, especially the latter, were indeed noble undertakings, whose vastness can only be conceived by those who are aware of the scarcity and dearness of manuscripts at that period of the world's history. M. Renouard has well compared its influence upon ancient civilization to that exercised by the invention of printing upon that of modern times. A city so munificently furnished with the appliances of literature, naturally became the resort of the learned, and the Ptolemies held out every possible inducement to them to take up their residence in so congenial a locality. No science received greater encouragement at their hands than that of Medicine, and the *School of Alexandria* quickly surpassed all others in celebrity; and, in the time of Galen, it sufficed to have studied there, or even to have only resided in the city, to obtain a reputation as a physician. Among the principal causes of the prosperity of the school may be named the encouragement given to the *dissection of the human body,* the Ptolemies themselves even wielding the scalpel. They likewise prided themselves upon forming valuable collections of animals and rare plants.

Unfortunately these desirable conditions for improvement were not to be of long continuance. Dissections were discontinued about the end of the second century, and natural researches became displaced by subtle and objectless discussions. The Roman Conquest produced an utter prohibition of dissection, for this people, stained with the bloodiest crimes, deemed the contact of a dead body an abominable profanation. The works of the professors of the Alexandrian School during its flourishing epoch are all lost, they being only known to us by tradition and by fragments preserved by writers of a later period, as Galen, Aræteus, Celsus, Pliny, and others. From these sources alone can we derive any idea of the state of medical science at the end of the second century of our æra —Galen, indeed, being our chief authority.

Several writings upon *Anatomy and Physiology* from the pen of Galen have descended to us, and manifest the vast progress the dissections permitted at Alexandria would in some measure entitle us to expect. To follow M. Renouard's detailed analysis is impossible, and we must content ourselves with his concluding remark.

"This sketch of the anatomy and physiology of Galen furnishes us with some idea of the immense progress that had been made during the anatomical period; and, when we consider that the greater portion belongs to the two first centuries of the foundation of the School of Alexandria, and is due chiefly to the labours of Herophilus and Erasistratus, we feel astonished at so rapid a development, as well as at the fortunate direction these two great physicians had impressed upon anatomico-physiological studies. Not only did they perform frequent dissections of the human body, but also had recourse to vivisections of animals. It is recorded, indeed, of Herophilus, that he did not hesitate to operate upon living criminals furnished him in the interest of science: but it is to be observed, that the fact is related by no contemporary, and was only known some centuries later as a popular tradition: so that it has been doubted by most historians, who observe that the same accusation has been falsely accredited, at different epochs, in reference to other celebrated anatomists.

"However, as we have already stated, the zeal for dissecting speedily cooled, and Galen only enumerates some five or six persons who occupied themselves with anatomy since the first Alexandrian anatomists, during the space of near 400 years which had intervened between that time and his own. None of these left a reputation anywise approaching that of Herophilus and Erasistratus; and Galen alone could sustain a comparison with them, by reason of his great number of experiments and anatomico-physiological discoveries. But in vain did he endeavour, by example and exhortation, to bring back his cotemporaries to the study of anatomy. He was unable to overcome their indifference. After his time, the employment of dissection seems to have become lost, whether from an increase of superstitious prejudices opposing it, or from the ignorant apathy of medical practitioners." P. 283.

Although *Hygiene* did not partake of the rapid progress which Anatomy and Physiology achieved, it did not either remain stationary, and Celsus, in his first book, presents us with the precepts which were most accredited in his time; and, if he adds but little to the materials derived from the Hippocratic Collection, at least he presents these with more order and concision. Galen also wrote much on hygiene, and insists upon the precepts to be observed in infancy and old age, and especially the former, to a much greater extent than his predecessors. He also lays great stress upon the influence of habit, and was the first who insisted that the due regulation of the passions should form a part of hygienic observances. Wishing, however, that the laws of hygiene should be deduced rather from preliminary determinations of the nature of man, the principles of which he is composed, &c., than from simple observation, the philosophical physicians fell into all kinds of ideal speculations and recommendations; and Galen has quite equalled his predecessors in the fastidious digressions he indulges in, so that his works on hygiene might be well reduced by four-fifths without any loss to their practical value.

*Pathology.*—In this period, under the influence of the Aristotle philosophy, writers distinguished the different branches of medical science with much more order and precision than heretofore; and, indeed, the love of method was carried to far too great an excess, so that Galen and others, by indulging in subtle and ill-defined distinctions, actually lost the greatest benefit—clearness—which arrangement bestows. The treatment of disease was now divided into hygienic, pharmaceutic, and chirurgical; but Leclere and Sprengel are certainly in error in supposing that each of these three departments of medical science were in the hands of as many classes of practitioners. As Peyrilhe observes, the distinction was a scholastic, not a civil one, for otherwise we ought to discern among the Romans, evidence of the existence of these three classes, while the separate existence of even one of them, the chirurgiens, properly so called, is not even established by minute investigation.

*Semeiology.*—We have already seen that the Hippocratic School studied symptoms not in reference to the disorder of any particular organ, but as an expression of the resistance made by the entire economy to the morbigenous principle. The study of such signs was carried to great perfection, as several of the works of that school testify. Moreover, towards the end of the former period, Praxagoras first drew attention to the connection between the state of the *pulse* and that of the economy—a subject to which little or no attention was paid in the Hippocratic works. In the present period, however, it excited much investigation, it being believed that variations in this indicated every variety of disease. Thus there was the pleuritic pulse, the suppurative, the phthisical, the hepatic, the splenic pulse, &c. &c. Galen's work upon the subject is divided into four sections, each comprising four books, in the first book of the first section no less than sixty varieties of the pulse being indicated! The inspection of the *urine*, next to the consideration of the pulse, most occupied the attention of the ancients, but the principal writings upon this subject were posterior to the time of Galen. Thus, the Hippocratists grouped together the most obvious symptoms of diseases and their events, and deduced thence their prognosis and diagnosis. The Anatomical School examined each symptom or order of symptoms apart, seeking the causes and signification of the minutest varieties they offered. "The synthetic mode of the former possessed grandeur and eclat, but was superficial and frequently at fault; while the analytical method of the latter, in affecting exactness and depth, was frequently trivial and subtle, losing itself amidst infinite littleness."

The *Nosography* of the Hippocratic School was exceedingly imperfect in the designation of specific differences in disease, owing to the absence of precise notions on anatomy and physiology, and its regarding symptoms rather as the expression of a general disorder than of particular organic lesions. During the Anatomical Period, however, Nosography attained a remarkable perfection, as may be seen by a perusal of the writings of Cælius Aurelianus and Aretæus, who both lived, according to the most probable opinion, in the second century of our æra. From the pen of each of these observers, we have a nearly complete treatise upon all the diseases observed in their times, forming precious monuments of ancient medicine. That of Aretæus, written in Greek, in an elegant and picturesque style, has procured for him the title of the most skilful depicter of disease among the ancients, while the work of Cælius, being written in bad Latin and disfigured by barbarous words of difficult interpretation, has prevented its contents obtaining the diffusion critics are unanimous in declaring they deserve. Galen also wrote at length upon all diseases, but his observations are so dispersed over different treatises, and overwhelmed by theoretical digressions, as to require a laborious examination and collation to be of much general service. The writers of this period minutely describe a variety of *chronic* affections, which those of the former wholly passed over; while, as regards acute disease, the Anatomical School also did much to perfect their diagnosis. The progress in this respect is exhibited by M. Renouard in transcribing the description of peripneumonia by Aretæus, and contrasting it with that of the Hippocratic Collection.

In *Therapeutics*, no new axiom was propounded, the Hippocratic doctrine of treating disease by its contraries still prevailing. For this reason great pains was taken to accurately determine its determining causes or essential phenomena, in order that remedies of the most opposite character might be applied. The Empirical sect, as we shall afterwards see, protested against this mode of procedure, declaring that the essential natures of diseases and of the primary action of curative agents were impenetrable, and reasoning founded upon them chimerical. The practical acquisitions in therapeutics during this period were however considerable. Aristotle had been the first to cultivate the science of Natural History, and Theophrastus, who inherited his manuscripts and museum, effected for botany what his master had accomplished for zoology. He described the physical qualities and medicinal properties of more than 500 species of plants. At Alexandria, large collections were also placed at the disposal of the learned, so that the *materia medica* soon became much enriched. Indeed, towards the end of this period, compound and exotic remedies had become so common as to lead to the most ridiculous polypharmacy. Then were invented the famous antidotes, the Mithridaciæ and Theriacæ, composed of from forty to sixty different ingredients, each being supposed to be gifted with its particular virtue. Galen, Pliny and Dioscorides, each attempted a classification of these numerous pharmaceutical substances, that of the last being most esteemed for its order, exactitude and clearness. "With an augmented *materia medica* and an improved diagnosis of disease, we may believe that the therapeutics of this period were more rational and efficacious than those of the former; and, in point of fact, the writings of Aretæus show us that, during the four centuries which separate the last Hippocratists from the epoch in which he lived, greater progress in respect to the treatment of some diseases had taken place than has been accomplished during the subsequent sixteen hundred years."

As might be expected, the dissections pursued by the Anatomical School contributed much to the advance of *Surgery,* and we accordingly find the chirurgical portions of the writings of Galen and Celsus incomparably superior to those of the Hippocratic school. *Celsus* seems to have lived towards the termination of the first century of the Christian æra, that is a little prior to Galen; and he treats various subjects not alluded to in the Hippocratic Collection, among which may be mentioned hernias, stone in the bladder, cataract, wounds of the abdomen, &c. The writings of the whole of the surgeons of the Alexandrian school are lost; and his work is therefore the earliest in which we find operative procedures described with clearness, method, and precision. M. Renouard places many passages from this writer in juxta-position with those treating upon the same topics from the Hippocratic Collection, and to his great advantage.

The Anatomical Period has furnished us with no *clinical histories* worthy of comparison with the Epidemics of Hippocrates.

> "Galen, who alone among the physicians of the period can sustain any comparison with that great man, has left us no collection of the sort. He only now and then relates the history of a patient with the evident object of exhibiting the superiority of his diagnosis and the excellence of his theory. We do not observe in his recitals, as in those of the painter of the epidemical constitutions, the plain and impartial historian, relating his facts without commentary; but we constantly meet with the prolix and subtle dialectician, who lets no phenomenon escape him without interpreting and lengthily discussing it. Many writers of the period, and Galen among others, commented upon the Hippocratic *Aphorisms;* but no one essayed the delivery of new ones. Most were, doubtless restrained by the respect paid to those ancient works whose reputation was colossal, others, perhaps, appreciated the abuse this species of literature—more pretentious than solid as respects medicine—is liable to. However this may be, we reproach not the writers of this period with having abandoned it." P. 316.

### *Theories and Systems.*

In proportion as the acquisitions in anatomy and physiology were augmented, the diagnosis of disease perfected, and therapeutical agencies increased, the necessity of classifying the masses of facts in some methodical order that they might be the more easily retained, and connecting them together by the most probable theory or explanation, was felt by many observers, and attempted by several of them. Neglecting all but capital differences however, the most important theories which have descended to us are four in number: Dogmatism, Empiricism, Methodism and Eclectism.

1. *On Dogmatism.*—The dogmatism taught at the School of Cos, supported by the approbation of Plato and Aristotle, had exerted its dominion over the medical world for several centuries when the School of Alexandria was founded, and the earliest professors of that school made little changes in it. The later ones made several modifications in these doctrines, but of all the sectarians who professed them the most fruitful, skilful and powerful, was

#### *Galen.*

Born at Pergamus, a town celebrated for its Æsculapian temple, its school of medicine, and its library, he received an excellent education, and profited by it with extraordinary assiduity. Already enabled to dispute with the most erudite in grammar, history, philosophy, and mathematics, he determined upon applying himself to the study of medicine. To this end, he travelled to many places, and resided some time at Alexandria. On his return, his great renown called him to Rome, where his brilliant language, profound erudition, and skilful practice conciliated to him the highest esteem; but where, too, his rapid success, his boasting, his undisguised disdain for his colleagues, also raised against him a powerful band of enemies who were the cause of his quitting the city—to return again, however, at the invitation of Marcus Aurelius; and he is supposed to have died there about the year 201. He declares himself a partisan of no sect, and ridicules those who acknowledge any of the then leaders of opinion. In fact, however, he explains, comments on, and amplifies the doctrines of Hippocrates, and refutes the objections of their adversaries. He thus states his own conception of his mission.

"No one before me has furnished the true method of treating disease. Hippocrates, I allow, had already pointed out the way; but, as he was the first to enter upon it, he could not proceed along it so far as was to be desired. The order he adopts is vicious. He omits certain important indications, and does not make all the necessary distinctions. After the manner of the ancients he is frequently obscure, for the sake of being concise. He says very little concerning complicated diseases. In a word, he has sketched out what another must complete; he has opened the route; it is for another to enlarge it, and render it easy of access."—*Methodus Medendi*, lib. ix.

Dr. Renouard gives an elaborate analysis of Galen's views in physics and physiology; but we think little or no advantage can accrue from our presenting an abstract of these to our readers. His elaboration of the doctrines of Hippocrates and Aristotle is ingenious, but subtle and minutely tedious; and, as a specimen, we may transcribe his opinions on the soul. Like Aristotle and Plato, he believed this to be composed of three portions, the vegetative, residing in the liver; the irascible, which has its seat in the heart; and the reasonable, which is placed in the brain. As instruments or servants of the soul, there are spirits generated in the liver, heart, and brain, besides which, each portion of the soul has certain secondary faculties at its disposal. Thus, the vegetative soul presides over the generative, augmentative, and nutritive faculties. The secondary faculties, again, are aided in their turn by a third order of faculties—the nutritive, whose principal seat is the stomach, being assisted by the attractive, retentive, assimilatory, and expulsive faculties. By the agency of such an hierarchy of souls, spirits, and faculties, Galen and his followers easily explained the various functions of the body.

In his *pathology* there is a similar assemblage of imaginary or abstract entities upon which acts and attributes are bestowed as if their existence were real. Some of the diseases are placed in the solids, others in the fluids, and others in the spirits. The action of medicinal substances is primary or consecutive. The former depending upon the predominant quality of the substance employed. One substance heats, because the element of fire predominates in it; and another cools, for the opposite reason. The combinations, degrees, and periods of production of these various effects are set forth in the most minute and elaborate manner. The consecutive action of, medicines is manifested after, and in consequence of the primary one. It varies much, some medicines opening the pores, others closing them ; some hardening, some softening, the tissues, &c. &c. Some secondary actions influence only certain organs or functions, and become diuretics, emmenagogues, &c.

"As to the principles which should guide the practitioner in the choice of therapeutical means applicable to each disease, the physician of Pergamus entirely adopts the axiom proclaimed by the School of Cos, which declares that diseases are to be cured by their contraries. Consequently, all his researches, all his pathological dissertations, tend to penetrate the essence of diseases, to render this apparent disengaged from all accidental accessories, in order that a treatment whose action is diametrically opposite might be applied. He makes this essence sometimes consist in an excess of one or more of the elementary qualities of the affected parts, sometimes in the reaction of the vital principle, the primary and efficient cause of all the symptoms. In that his doctrine is confounded with that of the dogmatists, and is nothing else than dogmatism itself, amplified, attenuated, and pushed to its remotest consequences. Sometimes, however, he takes into consideration the constriction or relaxation of the pores, which seems to resemble that of the Methodists. Lastly, in many passages, he professes a great consideration for the assemblages of symptoms and pure experience which the empirics place above every thing. Notwithstanding these slight and rare occasions, Galen has been, and ought to be, regarded as one of the columns of the Hippocratic dogmatism.

"Although he has written a great number of treatises on pathology and therapeutics, it would be difficult, if not impossible, to direct the treatment of any one malady by such a guide, so vicious is the order he has adopted, and so defective is his manner of treating his subject. I except from this proscription the four last books of his *De Locis Affectis*, in which he gives most excellent advice for the detection of the anatomical seat of diseases, especially mental and nervous ones. This treatise, joined to his writings on anatomy and physiology, constitute for him titles of imperishable glory, and justify or excuse the admiration, of which he was the object during more than twelve centuries.

"In respect to history, this encylopædical writer has also rendered immense services; for he has preserved for us the opinions of a great number of physicians whose works have perished, and especially of the chiefs of the sects. Thanks to him we are enabled to raise a corner of the veil which conceals the great struggles of the Dogmatists, Empirics, and Methodists. If the number of works which he has produced do not form a treasure easy of exploration by the practitioner, they are an arsenal whence the erudite and dialectician may draw arguments upon every description of medical question. We have approached a period when physicians pride themselves more by shining in the subtleties of dialectics and the display of a vain erudition, than in their practical wisdom: so that the very faults of this author secured the sceptre of medicine in his hand; for, as regards erudition, subtlety of reasoning, universatility of acquisitions, he only yields the palm to Aristotle, whom again he surpassed by the elegance, purity, and strength of his style. It is said that he wrote 500 volumes on medicine, and about half that number on other subjects. But it is to be observed that, among these volumes, some were only short manuscripts, consisting of a few pages." P. 335.

We are desirous of presenting our readers with a long, but interesting, extract from M. Malgaigne's Essay bearing upon this part of our subject. After adverting to Galen's proficiency in anatomy, his eager pursuit of experiments upon, and dissections of, animals, and his intimate acquaintance with the prevalent medical doctrines, M. Malgaigne observes—

"And yet no sooner does he commence his journey than he seems to take a pleasure in losing himself. In anatomy itself, that somewhat gross anatomy of the ancients, which only requires the eyes to see it, Galen sometimes turns away his eyes. Pre-occupied with final causes, he is so long searching for what ought to be, that he mistakes what is. If he professes an exalted admiration for Hippocrates, it is especially in reference to the works most unjustly attributed to that great master. Casting down without pity every obstacle, he elevates amidst their ruins the fundamental theory of the four humours constituting the human body, the blood, phlegm, bile, and atrabile; and, accordingly, as these humours are vitiated in quantity, quality, or mode of combination, you have the whole of pathology developed. To speak only of what relates to surgery, pure blood forms phlegmon, bile erysipelas, phlegm œdema, atrabile cancer, &c. Let the quality be vitiated, and from heated blood carbuncle will be produced; from a too-thick bile, phlegm, or atrabile, ulcer, scirrhus, or lepra is generated. Combine the humours in different degrees, the bile and the blood for example, and, according as one or other of these predominates, you have an inflammatory erysipelas, or an erysipelatous inflammation. Add to this, that the four principal qualities of the humours, cold, heat, dry, and humid, are found also in medicaments, so that fortunately we can combat the one by the other; and the theory is complete, intimately connecting pathology and therapeutics.

"How shall we explain that Galen, the anatomist, the experimenter, the observer and practitioner, has thus been led to adopt, strengthen, and cause the prevalence of a system so foreign to all positive observation? The explanation is found in the philosophical medium in which he lived. The philosophy of Plato, so long combatted by rival sects, gradually regained its ascendancy, and borrowed from Christianity a new power while lending it a useful support. Now, although Galen denies that he belongs exclusively to any school, it is to that of Plato he gives the preference. At an early period he had been instructed in all the subtleties of dialectics; and, like Plato, he assigned to this the first rank in the sciences, and would render it the earliest study of the physician. It is dialectics, he says, which teaches us the nature of bodies, what is formed of primary elements, and what of secondary ones. In searching for the elements the senses are powerless and useless, and we must interrogate the nature of things. It was then by a contemplation of the nature of things that he proceeded to the discovery of principles; these discovered, dialectics should suffice also to determine their applications; and we find in one of his books (*De Optimâ Sectâ*) this profession of philosophical facts, that no precept can be legitimate in medicine any more than in other sciences, but upon the triple condition of being true, useful, and in accordance with principles laid down. Strange principles these, which it was not sufficient that they should be true and useful! * * * * * * * * * The first essays of dogmatism then were produced under the direct influence of the philosophy of Plato, which also presided at its definitive establishment: and just as the medicine and surgery of Hippocrates sprang from the Socratic school, the medicine and surgery of Galen may be in good right termed Platonic. But the features of the one and the other were not alike. Hippocrates witnessed, while yet living, a revolt among his disciples: Galen made everything yield to the weight of his authority. Is this to say that error is more powerful than truth, and more easily seduces the thoughtless multitude? This is doubtless one of the saddest lessons taught by history. Nevertheless we must not judge Galen and his age with too much rigour. On the one hand, a true philosophy of the sciences as yet did not exist, and there was only, so to say, a choice of errors. On the other hand, minds were fatigued by that long anarchy which, during six ages, had buffetted them about; and they only sought a doctrine brilliant enough to rally them, and a master strong enough to rule over them. Galen presented himself opportunely. Gifted with a facility of conception, and an extraordinary love of labour, sharpened by hard studies in medicine and philosophy, practised in all the subtleties of dialectics, he overwhelmed his adversaries by the extent of his acquirements, the number of his productions, the force of his logic, the bitterness of his sarcasms, and, lastly, if we must admit it, the immensity of his pride. Add to this, that Hippocrates, resting upon incomplete and as yet uncertain observations, was menaced with an approaching and formidable struggle with the philosophy of pure reasoning, aided by all the eloquence of Plato. To Galen no peril of this sort presented itself. So far from this, the dogma of authority which he contributed to strengthen, and which was about to reign supreme in all the schools, assured to him, during a long succession of ages, a superiority without contest, and a domination without a rival."—*Bulletin,* p. 166.

2. *On Empiricism.*—As rival schools of philosophy to those of Plato and Aristotle were raised from time to time by Epicurus, Pyrrho, and others; so also the dogmatists in medicine found opponents who endeavoured to construct the medical art upon totally different foundations. The *Empirics,* as they were called, rose rapidly into estimation; one of their number, Heraclides of Tarentum, receiving the applauses of the leaders of opposite sects, Galen and Cælius Aurelianus. Unfortunately, their works are all lost, so that all accounts we have of them are derived from the writings of their adversaries. They employed three sources of medical instruction, which they termed the basis or tripod of Empiricism. These were the autopsy or personal observation, history or the study of observations collected by others, and epilogism or analogism, or the induction derivable from the two former sources.

A. *The Autopsy.*—The Empirics were so fully convinced of the truth of the axiom of Hippocrates, that experience is deceptive, that they instituted numerous precautions for guarding themselves as far as possible from sources of error; and laid down rules as minute to this end as any numerical enquirer of the present day would desire. Although they rejected all attempts at searching for the essential causes of disease, they did not believe all symptoms of equal importance, but measured them not by their pretended essence, but by sensible circumstances. They required, also, that the assemblage of symptoms should be considered together, and that, for due diagnosis, the condition of the entire economy, as far as possible, should be explored.

B. *History,* or the study of clinical relations contained in books is a valuable source of information, without which medicine must remain stationary. By its aid we participate in the experience of our predecessors, rectify our own observations, and fill up deficiencies in them. But, for books of this kind to be useful, we must be certain of their veracity, and among the characteristics of this are, the reputation of the author, similar accounts of the same fact delivered by observers of different times or places, and the agreement of the statements with our own observations.

C. *Epilogism or Analogism.*—If we have to treat a new or undescribed malady, neither of the above means of instruction can avail us, nor can they in the case of our having to treat a well-known affection while denied recourse to the indicated remedies. In these cases, analogy is to be our guide. If the disease has not been already described or treated, search for the mode of managing that one which nearest resembles it. If you have to substitute untried remedies for those of approved efficacy, let them at least as nearly resemble the latter as possible.

The Empirics substituted for the definitions of disease by the Dogmatists, according to their essential nature or proximate cause, simple descriptions, or abridged enumerations of their sensible qualities. They are said to have neglected the study of anatomy and physiology; but this, at least as regards anatomy, is an accusation we have only from their enemies, and one in flagrant contradiction with the fundamental basis of their doctrine: but, in respect to the physiology of the epoch, consisting, for the most part, in vague and futile dissertations upon the principle of life, the elements of the body, the generative cause, &c. &c. their neglect is somewhat excusable. In their *therapeutics* the Empirics were quite opposed to the maxim of diseases being cured only by their contraries. Believing the essential causes of disease to be impenetrable, they would not allow that any relation between this and the action of remedies was discoverable. Careful experience was with them the only recognised guide. As a treatment which has proved successful against a disease will exert again its efficacy in the event of its being employed in the same pathological condition, the essential point was to attain, by accurate description, the power of distinguishing this.

Empiricism has been erroneously, by some, considered a mere effect of the Pyrrhonean or sceptical philosophy, from which it differs in many essential points. It is evidently nearly allied to the philosophy founded upon the operation of the senses, of which Aristotle laid the first foundation, soon to desert the edifice he had commenced. The Empirics adopted the basis laid down by the great Perapatetic, but, instead of, like him, wandering about in search of generalities, mis-named principles, they were content to carefully collect and accurately describe particular facts, and to deduce practical rules from such observation. By never rising, however, to abstract generalities and universal axioms their work was left imperfect, and their final object not even indicated.

At first, Empiricism rapidly gained ground. Galen enumerates the names of many authors who professed it, and, at the time he wrote, the term had not become an injurious epithet; and he, who was never very measured in the terms he employed against his adversaries, and loaded the Methodists with those of contempt, speaks of the Empirics with much respect, and more than once admits being staggered by their reasoning, even while opposing it. The Methodist Aurelianus also speaks of them in honorable terms, and the Eclectic Celsus does so still more favourably.

"The circumstances amidst which the system was introduced were most favourable to its propagation. The medical theories, as we have seen, had fallen into confusion. All descriptions of principles, methods and opinions were questioned. The recent discoveries in anatomy, the introduction of a considerable quantity of new medicaments, whose properties were as yet undetermined, the always increasing fury of philosophical disputes—all had shaken the old dogmas without substituting any better in their place, or anything which received general assent. In the middle of such conjunctures, a doctrine which promised to put an end to the perpetual variations of dogmatism and to arrest the sterile incertitude of scepticism by fixing itself solely on the evidence of facts, would be welcomed with enthusiasm, especially by those practitioners whom daily experience had convinced them of the inutility of dialectics for the advancement of medicine.

"It was, however, not long before it was discovered that empiricism, though founded on simple observation, terminated neither differences of opinion or uncertainties of practice; for if rationalism, which proceeds from generals to particulars, is subject to deception, sensitism, or the experimental method, which proceeds from particulars to generals, has also its gropings. Besides the old Empirics, in stopping short at secondary generalities, without reaching first principles, or rather definite axioms, resembled workmen who ceased labouring in the midst of the erection of an edifice. Lastly, the greatest error on the part of Empirics, in the eyes of antiquity, was their not attaching themselves to any philosophical theory then known. Their doctrine might seduce practitioners by its apparent simplicity, but it could not satisfy speculative spirits. It possessed not, therefore, the conditions of life required by the learned world at that epoch. So was its fall complete. It remained cast down for ages, and its very name became synonymous with ignorance. Nevertheless, we shall see it rise again from this humiliation and aspire, even boldly, under the title of the experimental method, to an universal domination over the sciences, when the works of Bacon, Locke, and Condilliac shall have somewhat simplified its characteristics." P. 357.

3. *On Methodism.*—As in Empiricism each suite or combination of symptoms not hitherto described was deemed a new disease, and, as in the course of time, these species were constantly multiplying without any attempt at generalizing them being made, eventually the greatest confusion was produced; and a systematic mode of viewing science presented by Asclepiades, of Bythinia, a distinguished professor of Rhetoric at Rome, founded upon the philosophy of Epicurus, met with general favour. According to his views, the human body is formed of highly permeable tissues, perforated by invisible pores, through which pass and repass continually certain atoms of different shapes and volumes, incommutable, indivisible, and impalpable in their nature, being perceptible alone to the mind's eye. Health depended upon the exact symmetrical correspondence between the pores and the atomic molecules. However unsatisfactory his physiology, his therapeutics, from their simplicity, possessed a great attraction for his patients. His object was simply to enlarge the pores when too contracted, and *vice versa;* and his means of effecting it were chiefly of a hygienic character, such as various exercises, friction, &c. and the use of wine—to the exclusion of all remedies having a violent action. It is obvious that measures of this description could only prove useful in a limited class of affections, and accordingly Asclepiades' reputation was but ephemeral. His disciple, Themison, may be looked upon as the true founder of the Methodic sect. He divided acute and chronic diseases each into three genera, the constricted, fluxionary or relaxed, and the mixed—basing his distinctions not on the hypothesis of porosity, but on the sensible characters derivable from observation. Thus, *e. g.*, swelling, tension, induration, suppression of natural evacuations, were esteemed characters of the constricted genus. The classification of the species of disease under these genera was, however, of a very arbitrary character, diseases possessed of few or no characters in common, being grouped together; while different professors were at issue as to which genus certain maladies could be most appropriately referred. "Nevertheless, his essay at a pathological classification founded upon the obvious characters of diseases, and not on their occult or imaginary qualities, was a great step." The Methodists had but two *therapeutical indications* to fulfil: to relax when there was constriction, to constringe when there was flux or relaxation; and all their agencies were arrayed in one or other of these classes. Bleeding, emollient cataplasms, tepid laxative drinks, sudorifics, temperate air, sleep, moderate exercise, acted as relaxants. Darkness, cool air or drinks, red wine, vinegar, alum, &c. operated as astringents. They rejected all specific operation of medicines, such as purgative, diuretic, &c. excepting emetics, which, however, they did not give to evacuate bile or phlegm, but for the shock they imparted to the economy, opening and modifying the condition of the pores. The Methodists never troubled themselves with any researches for *causes*, whether occasional or proximate; since, from the moment these have taken effect, it is the disease induced by them which is to be cured; and it is from it, its nature, characters, and progress, and not from anterior circumstances, which no longer exert any influence, that indications must be drawn. They attached also little importance to a knowledge of the precise seat of the disease or part affected, nor to concomitant circumstances, as age, habits, general strength, season, &c., believing all such details superfluities which should not modify the treatment pursued. They discarded all the generalizations of the Dogmatists, and confined themselves as nearly as possible to an exact observation, forming morbid species of every different assemblages of symptoms. These multiplied at last so as to render their separate recognition a matter of difficulty; and, in classing them into generals the Methodists mistook the true object of such a proceeding. Instead of employing the generic distinctions as a means of more accurately determining the specific differences, they rejected the latter as useless, and confined their attention to the most general agreements. Thus, they would treat in the same manner mania, jaundice, amenorrhœa, because they belonged to the constrictive genus, &c. They paid no attention to the natural tendency of the vital forces, coction, or critical days; and they studied anatomy and physiology even less than the Empirics. So great was their desire for simplicity that all their patients were put upon a uniform system of regimen, in which the extent and duration of fasting were rigidly regulated.

So simple a method much facilitated the acquisition of a superficial knowledge of the medical art, and Thessalus boasted he could teach the whole science in six months. This doubtless contributed also to the rapid progress the sect at first made. It likewise satisfied the very prevalent love of generalization so characteristic of the epoch, and in some sort offered itself as a medium between the extremes of dogmatism and empiricism. With the former, it adopted rationalistic truths, but deduced from sensible phenomena; and, with the latter, it took observation for its guide, but unincumbered by a multitude of precepts. Galen was no dupe of the pretensions of the Methodists, but vigorously employed himself in unmasking the sophisms and in demonstrating the insufficiency of their theories and the dangers of their practice—bestowing on the professors themselves the epithets his satirical disposition suggested.

4. *On Eclectism.*—Each of the systems we have alluded to contain useful truths, which the reason and experience of all ages have confirmed, disfigured as is each by some grave error or exaggeration. Many physicians recognized this fact, and conceived a vague impression, that the truth is not to be exclusively found in any of these systems; but, not possessing the aid of the improved philosophy we employ, it was impossible for them to particularize what was good and what was evil in each. These practitioners, who called themselves *Eclectics* or *Episynthetics*, in order to shew that they adopted no system exclusively, but selected what was best from each, not possessing any general rule whereon to base their judgments, decided each particular question according to their taste, fancy, or reason. The habitual condition of an Eclectic was doubt, but not the doubt of the Pyrrhonist or Sceptic, which was as a principle absolute and universal, but one resulting from defective information on the point in question.

"While boasting that he chose that which each system offered of the best, he failed to indicate in what this last consists, and traced no rule for its recognition, but left this to the reason and experience of each individual; that is to say, he proclaimed individualism, doubt and isolation.

"In the midst of this conflict of theories, *dogmatism*, supported by the highest philosophical and medical celebrities, developed and maintained by Galen, offered the most extensive and most probable explanation of the living economy. It deserve to triumph, then, as it did in fact, its very errors contributing to its strength; for they harmonized with the predominant philosophy." P. 379.

### II. The Age of Transition.

[Commencing with the Death of Galen, A. D. 201, and terminating with the Revival of Letters, about A. D. 1400.]

### 5. *The Greek Period.*

[From the Death of Galen, A. D. 201, to the Destruction of the Alexandrian Library, 640.]

The entire civilized world has now submitted to the domination of the Roman Empire. No longer do the Schools of Greece resound with the discussions of her philosophers disputing upon the most arduous topics in morals, physics and metaphysics that can employ the mind and excite the imagination. In each department of the intellectual domain, some great name has obtained supremacy, and commanded a passive obedience; the true sense of his words, and not the discovery of truth or the establishment of principles, being the sole object of inquiry. In morals, Plato, Epicurus or Zeno, and in physics and metaphysics, Aristotle, reign supreme; and many centuries are yet to elapse before the authority of Galen in medicine is to meet with any rival. We have not now to chronicle the struggles of sects and systems. One sect and one system predominates, and the movement in medical science is slowly *retrograde.* One remarkable circumstance arrests, however, our attention: the sceptre of science is transferred from the hands of one people to those of another, and the language of Hippocrates and Galen is displaced by that of Avicenna and Albucasis. The first period in this Transition Age presents us with the writings of four Greek physicians, who had all studied in the School of Alexandria, this still maintaining a reputation until the Arabic invasion. Although they have in a great measure compiled from the writings of Galen and others, they have done good service to medical science, by enriching it with some new particulars, and preventing its lamp expiring amidst the general apathy.

1. *Celebrated Commentators.*—(1.) *Oribasius*, who lived in the fourth century, was the first author of importance noted in history after Galen. He wrote several works, of which only a portion remain. He freely avows his borrowing from prior authors, and especially Galen, the greater proportion of his materials; and he has the great merit of reproducing the ideas of others with so much clearness, order, and precision, that the abridgments which he furnishes are thus rendered more useful than the originals themselves. His writings upon anatomy are extensive, but consist exclusively in a reproduction of those of Galen; and so strong is his avowed predilection for that author, that he has so sensibly adopted his theoretical ideas, and even his expressions, as to have obtained for himself the cognomen of *Galen's ape*.

(2.) *Ætius* flourished towards the end of the fifth and beginning of the sixth century; he appears to have been the first medical practitioner of any importance who embraced Christianity; and, from his faith in the cure of diseases by miracle and invocation, he seems to have been a more ardent than enlightened professor of its doctrines. He possesses the same claims to our attention as Oribasius, having collected whatever he found remarkable in the writings of his predecessors into a body of doctrine in which nothing is omitted.

(3.) *Alexander* (*of Tralles*) enjoyed a brilliant reputation as a practitioner at Rome, and in his old age composed a book upon medicine. Like the foregoing, he professes a great admiration of Galen, but does not blindly adopt his opinions, at least as far as practice is concerned. There is much resemblance in the objects and execution of his work to that of Aretæus.

(4.) *Paulus Ægineta* probably lived about the end of the sixth and beginning of the seventh century. He acquired great celebrity, especially in midwifery—being the first practitioner, indeed, who rendered himself famous in the obstetrical department of medicine. He composed an abridgment of medicine in seven books, borrowing or even copying literally from his predecessors. In spite of his plagiarism, he is sometimes not destitute of originality, especially in the surgical portion of his book. He was evidently much accustomed to operations, many of which he describes more explicitly than Celsus, and, upon occasions, introduced the necessary modifications of these.

M. Malgaigne speaks much more disparagingly of this class of writers than does M. Renouard.

"The servitude to which the sciences were subjected was more oppressive in the Lower Empire than elsewhere. There were no longer observers or theorists. Compilers alone remained. Oribasius reduced the writings of Galen and others into seventy books, and then abridged this work into one of nine vols. Paulus Ægineta, in his turn, performed the office of abbreviator for Oribasius, and places before his work this humble and sad confession, 'that he had not taken up the pen to make original observations, seeing that the ancients had said all there was to say.' His only object was to prepare a convenient manual for those residing as a distance from large towns. Oribasius and Paulus had at least the merit of arrangement, but other compilers did not even take this trouble. Ætius prepared a work four times larger than that of Paulus by the use of the scissors, cutting out portions of prior writings, placing his scraps under certain chapters; and, descending still lower, Nicetas merely stitched entire treatises together, one after the other. He was not even a compiler, but a mere copyist.

"Without wishing to detract from Galen's just renown, it is evident that he was admirably served by circumstances, and that there is not much room for glorifying him over such disciples. When Henry Estienne, in the sixteenth century, assembled most of these authors into one collection, he placed on it the pompous title *Artis Medicæ Principes.* Assuredly a strange one, for they better merited the names of slaves than of princes. A calamity, not sufficiently to be deplored, has made their fortune. The original writings having perished, we feel happy in the possession of at least faint copies of them. From this loss it even results that the compilers, who shew some skill in their selections, are less precious to us than the mere copyists. Thus, for the exact history of surgery, Paulus Ægineta is very inferior to Ætius."—*Bulletin,* p. 168.

2. *Medical Organization.*—Upon examining in a general manner the medical organization of antiquity, we recognize *four distinct phases,* each corresponding to a particular condition of civilization. The first of these, occurring in the infancy of civilization, when the head of a family, or of a society, united in his person sovereign authority, the priesthood, and the secrets of science, may be termed *Patriarchal,* examples of it being found in the Jewish patriarchs and the Homeric heroes. (2.) When tribes had become united into nations, industry had made progress, and intellectual treasures accumulated, the memory of any one man proved insufficient for the retention of human acquisitions. Then the functions of authority were distributed in various modes, according to circumstances; but with the remarkable one of the rites of religion and the exercise of medicine being long retained in the same hands. This is to be observed in the history of the Egyptians, Hebrews and Greeks, and in that of widely-dispersed countries, at a certain epoch of their civilization, as ancient Gaul and Britain, China, Japan, the New World, &c. &c. It even prevailed in modern Europe, at the time when feudal anarchy displaced the Roman civilization. This almost universal coincidence is explicable by the fact that, in ages of ignorance and superstitious credulity, diseases were looked upon as a chastisement of Heaven, or the result of evil influence, rather than as springing from natural causes: so that prayers, exorcisms, expiations and sacrifices were at least as frequently resorted to as natural means of cure. The priesthood, participating in these delusions, considered the cure of disease as one of their attributes. This phasis of the profession may then properly be termed the *Sacerdotal.*

(3.) A time arrived, however, when medicine was no longer confined to the hands of the priesthood, and its occult secrets were laid open to the public. In Greece, this change commenced about the beginning of the fifth century B. C., after the dispersion of the Pythagoreans. No initiation or special preparation was now rendered peremptory. Science gained by the change, but the profession came to be regarded by the public as merely one of the means of money-getting. The change took place much later at Rome, for, until the time of Asclepiades of Bythinia, it was exercised under the patriarchal form, the priesthood not interfering with it more than any other classes. Cato the Censor much occupied himself with this species of domestic medicine, and pursued with the bitterest hatred those of the Greeks who practised professionally at Rome. The Greek physicians who resorted to Rome, however, by their intriguing, abandoned conduct and ignorance, justified many of his reproaches. Galen draws a most unfavourable picture of their manners and practices. No examination or proof of ability was demanded, and any impostor who chose usurped the title of physician. The excess of the evil, however, at last became insupportable and brought its own remedy. This third phasis may be termed the *Free Laical.*

(4.) The above-named abuses led to the interference of the legislative power, and a new phasis of the profession, which may be termed the *Legal* or *Organized Laical,* took place. The Emperor Antoninus Pius (A. D. 138—161) was the first to occupy himself with the subject, although we do not find that any true organization by means of examinations took place before the period of the Christian Emperors. After that period, the title *archiater,* which had formerly been used merely as a honorary appellation, and sometimes usurped by pretenders, became applied only to those who were attached to the Court as medical officers, or who, in the cities, had the duties of examining and admitting candidates to practice assigned to them. They were paid by the state, had many immunities, and treated the poor gratuitously. About the year 400 of our æra, a class of persons first employed themselves in the preparation of the *medicines* prescribed by the physicians, before which period these last prepared themselves, or with the aid of their disciples and servants, the remedies they required, as may be seen in several passages of Hippocrates and Galen. The *pharmacopoles* mentioned prior to this, were only druggists or herborists, from whom the physicians procured the simples necessary for the confection of their elaborate prescriptions.

3. *The Accessory Institutions of Medicine.*—Under this title M. Renouard comprehends hospitals, dispensaries, and the various other appliances for the treatment of the sick and infirm, and the instruction of the student. Neither the Greeks or Pagan Romans were provided with any institutions resembling these; and it is to Christianity we are indebted for their first establishment. A Roman Christian lady, Saint Paula, having, towards the end of the fourth century, repaired to Jerusalem, and founded there a religious society for the practice of good works, the sufferings of the pilgrims during sickness who repaired to that city, excited her sympathies, and induced her to open asylums for their succour. Emperors, Kings and Caliphs afterwards signalized their zeal by the erection of sumptuous edifices for the reception of the sick; and these establishments, at first founded in a merely philanthropic view, afterwards became admirable instruments for the profit of art and the advancement of science.

We will extract M. Renouard's general view of the period.

"In the time of Galen they only dissected animals, and this professor tells us that he made his anatomical demonstrations on apes, whose structure so much resembles that of man. Occasionally the physicians who followed the armies obtained permission to examine the body of some barbarian found on the field of battle; but eventually dissection was entirely discontinued, and the conformation of the human body only studied in books. The horror of the early

Christians at cadaveric researches was now more marked than that of the Pagans; and the Fathers of the Church launched their anathemas against any who should thus violate the remains of the dead. This abandonment of anatomy doubtless much contributed to the decay of the art of healing; but other causes powerfully concurred in favouring this. First among these we may place the rapid progress of Christianity, which disorganized the Pagan schools, discredited the profane sciences, ruined their teaching, and gave rise to a passion for religious controversy. Secondly, the small number of persons still attached to the culture of the natural sciences, trammelled by a vicious method, sought for the explanation of the phenomena of Nature only in the writings of the ancients, and, not daring to make any change in received doctrines, heavily toiled along the narrow path of the past.

"But, if this period was not fertile in scientific progress, it was so in social ameliorations. The commencement of the organization of medical teaching and practice was perhaps more profitable to humanity than a few additional discoveries in science would have proved; for the excesses of charlatanism had reached their utmost height. A law destined to place a bridle on these, by exacting certain conditions of skill and reputation in the aspirant, responded to an urgent necessity, and proved, as it always must, a public benefit. Lastly, the charitable institutions, of which this epoch furnished the first models, prepared a future, fertile in the sources of medical instruction. The first period then of the Age of Transition is not entirely profitless for the culture of science, and especially for humanity." P. 409.

### 6. *The Arabic Period.*

[From the Destruction of the Alexandrian Library, A.D. 640, to the End of the Fourteenth Century.]

Contrary to what might have been expected from the fanatical zeal with which the early Arab conquerors had destroyed the Alexandrian Library, many of their caliphs proved the most enlightened protectors and encouragers of literature; and that with a freedom from religious bigotry which induced the persecuted of other countries to resort to their courts for protection and improvement. The celebrity which many of the cities of Moorish Spain achieved likewise attracted students to them from all parts.

1. *Arabic Medicine.*—The first physician of eminence who wrote in the Arabic language was *Rhazes,* for many years a distinguished professor at Bagdad, and physician to the large hospital of that city. He seems to have acquired a rare success in the treatment of disease, and, from his vast experience, obtained the cognomen of "Experienced." He wrote much, but most of his works are lost. The principal one remaining, the *Continens,* a large compilation from various authors, made for his own use, would be of rare interest, did not the faithlessness of the versions, as M. Malgaigne observes, in which we have it, take much from its value. "Rhazes," adds this writer, "like Ætius, has preserved a multitude of the fragments of antiquity, which are only to be found in his collection; and, not content with drawing from so fertile a source, he furnishes us with many extracts from Indian and Persian manuscripts, which no one seems to have possessed subsequently to his time. In spite, too, of his submission to authority, we find, in some of his works, the signs of a man who has not renounced thinking and observing for himself." The Continens is moreover interesting as containing descriptions of new remedies introduced to the Arabs, and an account of the *eruptive fevers,* under the name of variolæ, first described by this people. This work enjoyed a great reputation among future Arab and even Latin writers, who freely employed its abundant materials, re-disposing them in better order, and correcting some of the errors. *Ali Abbas* rendered himself famous for the production of his *Almaleki,* which is chiefly extracted from the Continens.

*Avicenna,* surnamed the Prince of Physicians, was born in the year 980. His enthusiasm for study was immense, and he achieved for himself an immense reputation at Bagdad. His chief work, the *Canon,* was in fact, for five or six centuries, the classic book or medical code of Europe and Asia; and no author since Galen ever enjoyed so vast and durable an authority. The work is nevertheless nothing but a compilation from prior authors, especially Rhazes himself, as we have seen, a compiler from the Greeks and Romans. He takes Galen and Aristotle as his models, even surpassing them in subtlety and their other defects. His chapters on the *Eruptive* Fevers have however deservedly met with much approbation. He, as well as the other physicians of the period, recognized but two classes of these, the *variolæ,* or eruptions consisting in any description of pimple filled by fluid, as pustules, varioles, &c.; and *morbilli,* consisting of those in which the skin is covered with patches or spots, having scarcely any elevation, and containing no fluid. These last were termed *morbilies,* which signifies small disease or pestilence, as being of less gravity, and engendered by a smaller portion of morbigenous matter. At this period the eruptive fevers were classed with pests or epidemic pestilential fevers.

After the death of Avicenna, Arabic medicine declined, but recovered some portion of its renown in the hands of *Albucasis* in Spain, *Avenzoar,* and *Averrhœs,* during the course of the 12th century, afterwards to disappear for ever. *Albucasis* produced a work which, but a mere compilation, is still interesting for the description and drawings of surgical instruments it furnishes.

Although placed so much nearer the sources of antiquity than ourselves, the Arabs had more incomplete notions than we of its acquirements; for, notwithstanding they have preserved to us some fragments of the Greek authors now derivable from no other source, yet we have a far greater number of which they were ignorant. Moreover, they entirely neglected the Latin authors. Anatomy and physiology in their hands were retrograde; for, not themselves dissecting, they only copied the descriptions of Galen, adding to the original imperfections of the authors the additional ones of translation. Pathology, we have seen, was enriched by accurate descriptions of the external characters of the eruptive fevers; and Therapeutics made likewise some acquisitions by the substitution of mild aperients, as senna, cassia, and manna, for the drastics employed by the ancients. Many pharmaceutical and chemical improvements were likewise due to the Arabs, such as the preparation of syrups, spirits, distilled waters. Surgery also received some insignificant additions in the shape of unguents, plaisters, &c.—far from compensating it, however, for the abandonment of numerous operative procedures in use by the Greeks.

The utter extinction of Arabic medicine is thus explained by M. Malgaigne.

"Doubtless we must make great account of the decay of the nation itself; for civil wars, defeats, and invasions, are ill suited to the development of the sciences. But other powerful empires have become founded in Asia and Africa upon the ruins of the Arabic, having the same religion, manners, and language; and yet the schools have remained dumb, and the sciences have ceased to flourish. It is because the intolerance of Islamism, for some time softened down by the liberality of the Caliphs, re-appeared in all its violence under their successors. Two years after the death of Avicenna the Turks seized Persia, carrying with them all the fanaticism and barbarity of new converts; and Averrhœs, the last of the celebrated physicians of Spain, for having somewhat too liberally expressed his opinions, was deprived of his honours and prosperity, and forced to retract at the gates of a mosque, those present spitting in his face. We can understand why he had no successors."—*Bulletin,* 170.

2. *On Greek Medicine during the Arabic Period.*—While the Arab name was rising to the summit of the social and intellectual scale, that of the Greeks descended lower every day; and but one author, during a period of near 700 years, challenges the attention of the historian of medicine, that of *Actuarius,* who flourished in the 13th and 14th centuries. The last work from his pen which remains, *De Methodo Medendi,* is literally a reproduction of Galen's doctrines, but arranged with remarkable lucidity.

3. *On Latin Medicine during the Arabic Period.*—The disorganized condition of Western Europe during the dark ages, which constitute the larger portion of this period, although eloquently depicted by our author, is too familiarly known to require notice here. During the course of the 13th and 14th centuries the darkness began to dissipate, and a few men of genius and talents shone out from amidst the general mass of ignorance and superstition. The writings of *Dante, Petrarch,* and *Boccacio,* revived the good taste and pure language of the learned ages, while *Leonardo of Pisa* introduced a knowledge of the Arabic figures and algebraic characters. Amid the profitless scholastic and theological discussions which alone possessed excitement for the learned men of the period, *Roger Bacon,* so much in advance of his times, sent forth those wonderful previsions of scientific reform and future discovery, which ensured for him the persecution of his cotemporaries, and the admiration of posterity.

4. *Medical Organization.*—We have already described the commencement of medical organization in the empire prior to the destruction of the Alexandrian School, and it is probable that this was maintained in the Greek empire of Constantinople. As to the countries submitted to the Arabs, we know, in many cities, schools and academies were founded for teaching and improving the medical art; but of the nature of their government, and their mode of dealing with candidates, we have no account.

[*We had at first intended to have divided this article into two portions for successive numbers of the Journal; but believing, upon reconsideration, that the interest of the subject will be best maintained by presenting it entire and complete at once, we have resumed our analysis of M. Renouard's most interesting volume at page* 399 *of the present Number.*]

